# Rapid and sensitive real-time assay for the detection of respiratory syncytial virus using RT-SIBA®

**DOI:** 10.1186/s12879-017-2227-x

**Published:** 2017-02-10

**Authors:** Kevin E. Eboigbodin, Kirsi Moilanen, Sonja Elf, Mark Hoser

**Affiliations:** 1 Research and Development, Orion Diagnostica Oy, P. O. BOX 83, FI-02101 Espoo, Finland; 2 Molecular Biology, GeneForm Technologies, Broadstairs, UK

## Abstract

**Background:**

Respiratory syncytial virus (RSV) is one of the most common causes of respiratory tract infections among young children and the elderly. Timely and accurate diagnosis of respiratory tract infections improves patient care and minimizes unnecessary prescriptions of antibiotics. We sought to develop a rapid nucleic acid tests for the detection of RSV within minutes, while retaining the high sensitivity achieved with RT-PCR.

**Methods:**

We developed and evaluated a reverse transcription isothermal nucleic acid amplification method, reverse transcription strand invasion based amplification (RT-SIBA), for the rapid detection of RSV.

**Results:**

The developed RT-SIBA assay showed good sensitivity by detecting as few as 10 copies of RSV RNA within 20 min compared with reverse transcription polymerase chain reaction, which took approximately 2 h. The performance of the RT-SIBA RSV assay was further investigated using nasopharyngeal swab specimens. The RT-SIBA assay had a sensitivity of 100% (25/25) and a specificity of 100% (15/15).

**Conclusion:**

RT-SIBA did not require highly purified RNA for the rapid detection of RSV and was therefore compatible with rapid specimen processing methods. This reduces the complexity of specimen preparation and further shortens the total amount of time needed to detect RSV in clinical specimens. The developed RT-SIBA assay for RSV could be a useful tool for prompt management of this infection

## Background

Respiratory syncytial virus (RSV) is a leading cause of respiratory tract infections and is often associated with high morbidity and mortality rates among children as well as the elderly and immunocompromised patients [1, 2]. RSV accounts for more than 60% of acute respiratory tract infection cases among young children. For children younger than 1 year, RSV can account for approximately 80% of lower respiratory infections, and almost all children are infected by RSV before the age of 3 years [3, 4]. RSV is a member of the *Paramyxoviridae* family and harbors non-segmented negative-sense single-stranded RNA as its genetic material. There are two major RSV subtypes, RSV-A and RSV-B, which are distinguished by their surface glycoproteins and genetic variation [5]. The two RSV subtypes often co-circulate during epidemic seasons [6, 7].

Timely and accurate diagnosis of RSV infections plays an important role in their management and treatment because it is often challenging to distinguish RSV infections from other viral or bacterial respiratory tract infections based on the clinical presentation alone [8]. Timely diagnosis also plays a crucial role in avoiding inappropriate use of antibiotics, thereby lowering the risk of antimicrobial resistance [9]. Nucleic acid amplification tests (NAATs), especially reverse transcription polymerase chain reaction (RT-PCR), are increasingly used to detect RSV because they are more sensitive than rapid antigen detection tests (RADTs) [10]. Despite the superior sensitivity of RT-PCR over RADTs, RADTs remain an important diagnostic tool, especially in point-of-care settings, due to their shorter turnaround time and ease of use [11, 12]. Conversely, specialised training and sophisticated and costly instrumentation are required to perform RT-PCR. Consequently, the method is often confined to specialised and large central laboratories.

In this study, we sought to develop a NAAT for RSV that has a similar time-to-detection as RADTs, while retaining the high sensitivity achieved with RT-PCR. To reduce the diagnostic time, we developed an isothermal reverse transcription strand invasion based amplification (RT-SIBA) assay for the detection of RSV. In RT-SIBA reactions, RSV RNA is first transcribed to cDNA by a reverse transcriptase and immediately amplified and detected under isothermal reaction conditions [13, 14]. SIBA utilize a recombinase-coated single-stranded invasion oligonucleotide (IO) for the separation of a target duplex. This leads to the generation of a single-stranded target template, which subsequently becomes bound and extended by target-specific primers via the action of polymerase. The repeated cycles of recombinase-dependent strand separation and subsequent polymerase-dependent extension of the target leads to an exponential amplification under isothermal conditions.

## Methods

### Clinical specimens and rapid processing

A total of 40 nasopharyngeal (NP) swab specimens were obtained from Discovery Life Sciences Biobank (Discovery Life Sciences, Inc., CA, USA). The specimens were previously determined to be positive (*n* = 25) or negative (*n* = 15) for RSV using the Alere BinaxNOW RSV , a rapid immunoassay test (Alere Inc, USA). These specimens were used to evaluate the performance of RT-SIBA in comparison with previously published RT-PCR for the detection of RSV [15]. Viral RNA was extracted from the specimens using the QIAamp Viral RNA Mini Kit (Qiagen, Germany) according to the manufacturer’s instructions. Thereafter, 2 μl of purified RNA was added to the RT-SIBA or RT-PCR RSV assay. A set of NP swab specimens was also subjected to a quick and crude specimen preparation protocol instead of RNA purification. To this end, 5 μl of the NP swab specimen was added to 45 μl of lysis buffer (2% Triton X-100 and 125 mM magnesium acetate; Sigma-Aldrich, USA), and 2 μl of the crude lysate was subsequently added to the RT-SIBA reaction.

### Preparation of RSV RNA standards

RNA was purified from RSV-A (ATCC VR-1540) and RSV-B (ATCC VR-1400) American Type Culture Collection (LGC Standards, Germany) viral particles using the QIAamp Viral RNA Mini Kit (Qiagen) according to the manufacturer’s instructions. The purified RNA was quantified using the Genesig® RSV RT-PCR kit (Primerdesign™ Ltd., UK). The kit includes RSV RNA standards with pre-determined copy numbers. These standards were used to generate standard curves for the quantification of RSV-A RNA and RSV-B RNA. RT-PCR was performed on a Bio-Rad qPCR CFX95 instrument (Bio-Rad Laboratories, CA, USA). The quantified RSV RNA was subsequently used to establish the analytical sensitivity of the RT-SIBA RSV assay in comparison with a previously published RT-PCR assay for the detection of RSV [15].

### Design of the RT-SIBA RSV assay

The assay was designed to detect all known human RSV strains including subtypes A and B [14]. RSV sequences were retrieved from the Virus Pathogen Resource [16] as well as the National Center for Biotechnology Information database and aligned. The assay was designed to amplify ~70 nucleotides within the highly conserved region of the RSV N-protein gene. The assay used the same target region within the RSV-A and RSV-B genomes. The RSV-A and RSV-B genomes share about 90% homology within the chosen target region. A set of primers and invasion oligonucleotides (IOs) were designed to amplify this target region. The RSV assay used one forward primer, one reverse primer, two IOs, and one probe. The use of multiple IOs facilitated improved detection of RSV-A and RSV-B in the same reaction tube, but could not differentiate between the two subtypes of RSV. The RT-SIBA assay was also developed to detect the bacteriophage MS2. Target-specific probes for both the RSV and MS2 assays were designed as previously described [17]. This allowed the RSV and MS2 assays to be multiplexed in the same reaction tube. The MS2 assay served as an internal control for monitoring sample-derived inhibition [18].

### RT-SIBA RSV assay

RT-SIBA reactions were performed using the SIBA® reagent kit (Orion Diagnostica Oy, Finland) with the addition of 16 units of GoScript™ Reverse Transcriptase (Promega, UK). The RSV forward and reverse primers were each used at a concentration of 400 nM. RSV-A, RSV-B, and MS2 IOs were used at concentrations of 200, 200, and 100 nM, respectively. The RSV and MS2 probes were each used at a concentration of 100 nM. The sequences of the oligonucleotides used for the RT-SIBA RSV and MS2 assays are shown in Table 1. In total, 2000 copies of MS2 RNA (Sigma-Aldrich) were added to the reaction. The UvsX and gp32 enzymes were used at a concentration of 0.25 mg/ml. In total, 2 μl of the sample or RNA template was used in a total reaction volume of 20 μl. RT-SIBA was detected using fluorophore-labeled probes and SYBR Green 1 (1:100,000 dilution). The reactions were incubated at 41 °C for 60 min, and fluorescence readings were taken at 60 s intervals using Bio-Rad CFX 96 (Bio-Rad Laboratories). A melting curve profile was generated after amplification at 40–95 °C to verify that the reaction products were specific.

**Table 1:** RT-SIBA oligonucleotides used in this study

Name	Sequence 5′ -- > 3′
RSV-F primer	GAGCCACCTCTCCCAT
RSV-R primer	AGTCAACATTGAGATA
RSV-A IO	**CCCCC** TTTCTTTTAGCATTTTTTTGTAGGATTT mUmCmUmAmG mAmUmUmCmUmA
RSV-B IO	**CCCCC** TCTCTTTTAGCATTTTTTTGTAGGACTT mUmCmUmAmG mAmUmUmCmUmA
RSV probe	/56-ROXN/+CA + A + TA + T + T + GA + GA + TA/3IABkFQ/
MS2-F primer	CATGATATTCTGGGCAATAGT
MS2-R primer	CGTAGATGCCTATGGTTC
MS2 IO	**CCCCCCCCCC** AATAGTCAAATCGACCCAAATCCATTTT mGmGmUmAmA mCmGmCmCmGmGmAmAmC
MS2 probe	/5CY5/A + TA + TT + CT + GG + GC+ A + A/3IAbRQSp/

For invasion oligonucleotides (IOs), bold sequences denote non-homologous seeding area sequences. mA, mC, mG, and mU denote 2′-O-methyl RNA nucleotides. *F* forward, *R* reverse, *+* locked nucleic acid bases, *IABkFQ* Iowa Black FQ quencher, *IAbRQSp* Iowa Black RQ quencher, *RSV* respiratory syncytial virus, *RT-SIBA* reverse transcription strand invasion based amplification. The RT-SIBA RSV assay was compared with a previously published reverse transcription polymerase chain reaction RSV assay (12)

### RT-qPCR

The performance of the RT-SIBA assay was compared with a previously published RT-PCR assay for the detection of RSV-A and RSV-B [15]. Both RT-SIBA and RT-PCR reactions were performed at the same time. 2 μl of the sample or RNA template was used in a total RT-PCR reaction volume of 20 μl. The concentrations of the probes and primers were previously described [15]. RT-PCR was performed using EXPRESS qPCR SuperMix (Thermo Fisher Scientific, USA). A thermal program of 50 °C for 15 min, 95 °C for 2 min, and 45 cycles of 95 °C for 30 s and 45 °C for 1 min was used with Bio-Rad qPCR equipment (Bio-Rad Laboratories).

## Results

### Analytical sensitivity of the RT-SIBA RSV assay in comparison with RT-PCR

The analytical sensitivity of the RT-SIBA RSV assay was compared with that of the previously published RT-PCR RSV assay [15]. The sensitivities of the RT-SIBA and RT-PCR assays for RSV were evaluated in three independent experiments by adding serial dilutions of RNA extracted from RSV, with 10–10^5^ copies per reaction. Five replicates of each dilution were used. RT-SIBA reactions were detected using both target-specific probes and SYBR Green. The RSV assay included the MS2 internal control to assess sample-derived inhibition. The RSV probe was labeled with ROX and Iowa Black FQ quencher, whereas the MS2 probe was labeled with Cy5 and Iowa Black RQ quencher.

**Fig. 1:**
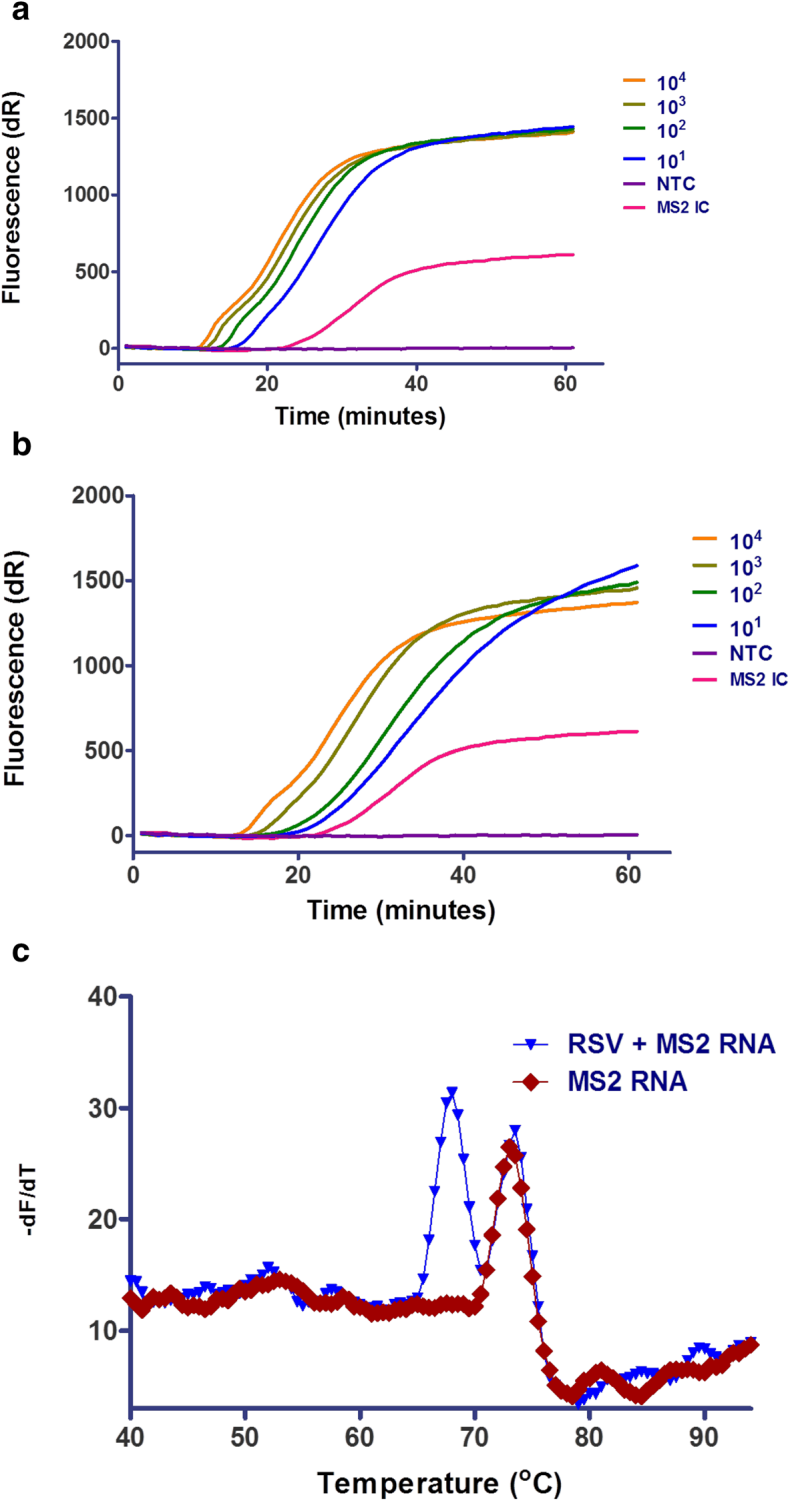
Sensitivity of RT-SIBA for the detection of respiratory syncytial virus (RSV). **a** RSV-A RNA and **b** RSV-B RNA. **c** Melting curve profiles of RSV (1000 copies of RSV-A RNA) and MS2 RNA in the same reaction tube. MS2 was used as the internal control (IC, 2000 copies per reaction). No template control (NTC)

RT-PCR incorporated a target-specific probe, which enabled specific detection of RSV-A and RSV-B subtypes. The results are shown in Fig. 1 and Table 2. Both RT-SIBA and RT-PCR reliably detected as few as 10 copies of RSV RNA (5/5 replicates in every control dilution). The average amount of time taken to achieve positive reactions in RT-SIBA was compared with the corresponding cycle threshold (ct) values for RT-PCR (Table 2). RT-SIBA reactions were performed at a constant temperature and consequently did not require thermal cycling. Hence, RT-SIBA data were collected at 1 min intervals. RT-SIBA RSV assays detected 100 copies of RSV-A and RSV-B at approximately 14 and 16 min, respectively. The corresponding ct values for 100 copies of RSV-A and RSV-B RNA were approximately 27 and 30, respectively. The corresponding average amount of time taken to achieve positive reactions with RT-PCR can be around 2 h due to the initial reverse transcription step as well as the instrument ramp time. RSV was detected faster by RT-SIBA than by RT-PCR.

**Table 2:** Analytical sensitivity and average detection time of RT-SIBA compared with those of RT-PCR for the detection of RSV

	RT-SIBA Average amount of time taken to achieve positive results (minutes)		RT-PCR Average cycle threshold to achieve positive results (ct)	
RSV RNA copy number per reaction	RSV-A	RSV-B	RSV-A	RSV-B
10^4^	11	13	21	20
10^3^	12	14	24	23
10^2^	14	16	27	26
10^1^	16	20	31	30
0	ND	ND	ND	ND

*ND* not determined, *RSV* respiratory syncytial virus, *RT-PCR* reverse transcription polymerase chain reaction, *RT-SIBA* reverse transcription strand invasion based amplification

### Analytical specificity of the RT-SIBA RSV assay

Subsequent melting curve analysis revealed the presence of a single specific amplicon in reactions containing RSV RNA. The RT-SIBA RSV assay did not produce any detectable amplification signal in the absence of RSV RNA (no template controls). This was also the case when related nucleic acids from other common respiratory pathogens were added to the RT-SIBA RSV reactions (adenovirus, influenza A, influenza B, rhinovirus, coronavirus, *Staphylococcus aureus*, and *Streptococcus pyogenes*). These findings, together with the negative sample panel, showed the specificity of the RT-SIBA RSV assay.

### Performance of RT-SIBA RSV with NP swab specimens

The performance of RT-SIBA for the detection of RSV was further evaluated using NP swab specimens. A total of 40 NP specimens were used, 15 of which were negative for RSV and the other 25 were positive for RSV, as previously determined by the Alere BinaxNOW RSV test. Total RNA were extracted from these specimens and subsequently used to further evaluate the performance of the RT-SIBA and RT-PCR RSV assays. The results are shown in Table 3. Both the RT-SIBA and RT-PCR RSV assays detected RSV in all 25 NP specimens that were previously confirmed to be positive for RSV. Conversely, no amplification reaction was detected by the RT-SIBA RSV assay with the 25 NP specimens that were previously confirmed to be negative for RSV. The RT-SIBA RSV assay detected RSV in positive specimens within approximately 20 min, while the detection time of RT-PCR RSV was approximately 2 h. The faster detection time is one advantage of RT-SIBA over RT-PCR.

**Table 3:** Performance of RT-SIBA and RT-PCR for the detection of RSV from NP swab specimens using purified RNA or the rapid lysis protocol

		Extracted RNA from clinical specimens		Rapid sample processing
Sample no.	Original sample type	RT-SIBA detection time (minutes)	RT-PCR, cycle threshold Ct (result)	RT-SIBA detection time (minutes)
1	NP UTM	10	25 (RSV B+)	16
2	NP UTM	11	27 (RSV B+)	31
3	NP UTM	11	27 (RSV B+)	19
4	NP UTM	10	24 (RSV B+)	22
5	NP UTM	11	26 (RSV B+)	15
6	NP UTM	11	27 (RSV B+)	17
7	NP UTM	11	26 (RSV B+)	15
8	NP UTM	10	23 (RSV B+)	16
9	NP UTM	11	28 (RSV B+)	44
10	NP UTM	12	29 (RSV B+)	20
11	NP UTM	10	23 (RSV B+)	16
12	NP UTM	20	25 (RSV B+)	36
13	NP UTM	14	28 (RSV B+)	35
14	NP UTM	11	28 (RSV B+)	16
15	NP UTM	12	28 (RSV B+)	20
16	NP UTM	10	24 (RSV B+)	19
17	NP UTM	12	26 (RSV A+)	17
18	NP UTM	11	25 (RSV B+)	21
19	NP UTM	11	25 (RSV B+)	16
20	NP UTM	11	33 (RSV B+)	15
21	NP UTM	20	27 (RSV B+)	37
22	NP UTM	10	25 (RSV B+)	15
23	NP VTM	14	31 (RSV A+)	22
24	NP VTM	12	27 (RSV A+)	15
25	NP VTM	13	31 (RSV B+)	21
26–40	NP UTM	ND	RSV (−)	ND

*ND* not determined, *NP* nasopharyngeal, *RSV* respiratory syncytial virus, *RT-PCR* reverse transcription polymerase chain reaction, *RT-SIBA* reverse transcription strand invasion based amplification, *UTM* universal transport medium, *VTM* viral transport medium

### Rapid specimen processing

We evaluated the possibility of performing one-step rapid lysis of NP specimens, instead of purifying RNA from them. This reduces the complexity of specimen preparation and could further shorten the total amount of time needed to detect RSV from clinical samples. The results are shown in Table 3. RSV was detected in all 25 RSV-positive NP specimens by RT-SIBA using this crude sample lysis method. The detection of RSV by RT-SIBA was faster with clinical specimens extracted using a commercial extraction kit than with clinical specimens subjected to the rapid sample processing method. This could be due to a difference in the final amount of RNA present in the reaction. RSV was not detected in any of the RSV-negative NP specimens by the RT-SIBA RSV assay. It took approximately 5 min per specimen to perform the rapid specimen preparation protocol, whereas purification of RNA from NP specimens took approximately 45 min. The results suggest that a crude sample processing protocol could be used with RT-SIBA to further shorten the total amount of time needed to detect RSV from NP specimens.

## Discussion

In this study, we developed and evaluated an alternative NAAT, RT-SIBA, for the rapid detection of RSV from NP swab specimens. This method is reported to be useful for the detection of pathogens [13, 14, 17, 19]. SIBA is an isothermal nucleic acid amplification method that relies on a recombinase-dependent oligonucleotide and target-specific primers to rapidly amplify a DNA duplex [14, 17]. The inclusion of a reverse transcription enzyme in SIBA reactions (RT-SIBA) facilitates the rapid detection of RNA from pathogens [13]. SIBA reactions are performed at a low and constant temperature (41 °C) and therefore do not require complex instrumentation, in contrast with RT-PCR. Consequently, RT-SIBA is compatible with less complex devices, which could be suitable for point-of-care applications. These portable devices do not require highly specialised personnel to run and interpret the results [19, 20]. In this study, RT-SIBA reactions were performed with a qPCR device for the convenience of performing multiple reaction conditions and replicates. The RT-SIBA assay was developed to detect both the RSV-A and RSV-B subtypes, but not to distinguish between them.

The developed RT-SIBA RSV assay displayed a similar analytical sensitivity and specificity as the RT-PCR method for the detection of RSV. The RT-SIBA assay detected 100 copies of RSV-A and RSV-B RNA after 14 and 16 min, respectively. The detection time differed between the RSV subtypes because the RSV-B target region has two nucleotides that are not complementary to the assay. SIBA can typically tolerate 1–3 nucleotide mutations in the assay target region, but this leads to a slightly slower detection time, as seen with RSV-B RNA [14]. Nonetheless, the detection time reported for RT-SIBA RSV was significantly faster than that of RT-PCR, which took approximately 2 h. RT-SIBA detected RSV in a highly specific manner, without cross-reacting with other common respiratory pathogens.

The performance of the RT-SIBA RSV assay was further verified using 40 NP swab specimens. RT-SIBA detected RSV from all NP specimens that were previously determined to be RSV-positive by RT-PCR. Based on the number of NP samples used, the RT-SIBA assay displayed a sensitivity of 100% (25/25) and a specificity of 100% (15/15). The method was compatible with a rapid sample processing method, further shortening the total amount of time needed to detect RSV from clinical specimens. The detection times of RT-SIBA are similar to those of RADTs. RADTs used to detect RSV can take 15–30 min [21, 22]. However, RADTs are often less sensitive than RT-PCR [10]. Despite the superior sensitivity of RT-PCR over RADTs, RADTs still play an important role in the diagnosis of RSV, especially within point-of-care settings, due to their rapid detection time and ease of use. RT-SIBA displayed a detection time as rapid as that of RADTs and a high analytical sensitivity similar to that of RT-PCR.

## Conclusion

In conclusion, the developed RT-SIBA for the rapid detection of RSV displayed high levels of sensitivity and specificity for RSV RNA. The method significantly shortens the total detection time needed for the diagnosis of RSV from nasopharyngeal swab specimens. RT-SIBA is compatible with rapid specimen processing, a portable device, and is therefore a useful molecular diagnostics tool for the rapid diagnosis of RSV infections especially within the point of care settings.

## Abbreviations

NAATs: Nucleic acid amplification tests
RADTs: Rapid antigen detection tests
RSV: Respiratory syncytial virus
RT-PCR: Real time polymerase chain reaction
RT-SIBA: Reverse transcription strand invasion based amplification
